# Prevalence and Age Structure of Polypharmacy in Poland: Results of the Analysis of the National Real-World Database of 38 Million Citizens

**DOI:** 10.3389/fphar.2021.655364

**Published:** 2021-04-15

**Authors:** Przemysław Kardas, Filip Urbański, Aneta Lichwierowicz, Ewa Chudzyńska, Grzegorz Kardas, Marcin Czech

**Affiliations:** ^1^ Department of Family Medicine, Medical University of Lodz, Łódź, Poland; ^2^ National Health Fund, Warsaw, Poland; ^3^ Department of Internal Diseases, Asthma and Allergy, Medical University of Lodz, Łódź, Poland; ^4^ Department of Pharmacoeconomics, Institute of Mother and Child, Warsaw, Poland

**Keywords:** polypharmacy, prevalence, national cohort, pharmacoepidemiology, Poland, retrospective studies

## Abstract

**Introduction:** Polypharmacy is a risk factor for adverse health outcomes, higher use of medical services and additional costs. The problem has gained attention as a consequence of aging and related multimorbidity. Therefore, there is an urgent need to adopt effective interventions aimed at reducing its burden. In order to achieve this, in-depth understanding of the prevalence of polypharmacy is required. Of particular interest is, however, assessing prevalence of polypharmacy in various age groups, to reach the right target for these interventions. So far, only limited data on polypharmacy among non-elderly individuals have been available.

**Aim of study:** To assess overall prevalence of polypharmacy in Poland as well as its distribution in various age groups using real-world data.

**Methodology:** A retrospective analysis of complete dispensation data of national payer organization for the years 2018–2019. The analyzed dataset included data on dispensation of reimbursed drugs, and exclusively for 2019, also non-reimbursed drugs. Polypharmacy was defined as dispensation of ≥5 prescription medications within six months.

**Results:** In the analyzed national cohort of 38 million Polish citizens, the prevalence of polypharmacy was found to be 11.7% in 2018 and 11.6% in 2019. With age, the prevalence of polypharmacy increased, reaching the value of 56.0% in those aged 80+ in 2018, and 55.0% in 2019. Altogether, among those aged 65+, the polypharmacy was present in 43.1% in 2018, and 42.1% in 2019. In the youngest group of citizens, i.e., among those aged below 20 years, polypharmacy was found in 0.9%, and 0.8% in 2018 and 2019, respectively. Prevalence of polypharmacy, calculated for 2019 according to dispensation of five or more reimbursed and non-reimbursed drugs for the whole Polish population, was 21.8% for January-June, and 22.4% for July-December 2019. Among those aged 65+, the relevant numbers were 62.3%, and 62.9%, respectively.

**Conclusion:** This study, being the first nationwide assessment of polypharmacy in Poland, confirmed its high prevalence. We found polypharmacy present in over one fifth of Polish society. Peaking in the elderly, polypharmacy occurred in each age group. These results lay the foundations for future interventions focused on reducing the scope of this problem in Poland.

## Introduction

Polypharmacy is a term describing a scenario in which multiple medicines are prescribed to the same patient. Since the term has not been provided with one standard definition, it is most often described as concomitant use of five or more drugs (Masnoon et al., 2017; Khezrian et al., 2020). Of course, polypharmacy is not always a wrong strategy as more complex patients may benefit from what is referred to as ‘appropriate polypharmacy’ (Hughes, 2020). However, polypharmacy entails a higher risk of medication non-adherence. Studies have shown that increasing a number of drugs taken by a patient leads to a higher probability of non‐adherence by up to 16% for each additional drug (Gray et al., 2001). Moreover, polypharmacy favors potentially inappropriate prescribing and all its negative clinical consequences (Lee et al., 2020). The so-called ‘inappropriate polypharmacy’ often leads to increased risk of drug-drug interactions, toxicity and other adverse drug events (Scondotto et al., 2018). As compared to people taking from two to four drugs, the percentage of patients exposed to potential interactions among those taking at least 15 drugs increases from 10 to 81% (Guthrie et al., 2015). The most important clinical consequences of polypharmacy include higher morbidity, with particular increase of risk of geriatric syndromes (such as e.g., cognitive impairment or falls), and higher mortality (Davies et al., 2020). Moreover, polypharmacy has been attributed as a risk factor of frailty in elderly (Gutiérrez-Valencia et al., 2018). All these, in turn, lead to profound consequences at the population level, i.e. increased use of healthcare services, higher risk of hospitalization and institutionalization, and much greater health-associated costs (Maher et al., 2014; Wastesson et al., 2018).

In the light of the above, the importance of polypharmacy for public health is indisputable. Therefore, it is considered to be ‘one of the greatest prescribing challenges’ (Payne and Avery, 2011). This challenge is even growing due to the rapid rise in its global prevalence that has been observed recently, to a large extent being caused by two interlinked factors, aging and multimorbidity. (Hovstadius et al., 2010; Charlesworth et al., 2015; Craftman et al., 2016; Martin-Pérez et al., 2017; Zhang et al., 2020) (Carmona-Torres et al., 2018).

Effective curative (e.g., antibiotics) and preventive (e.g., vaccinations, lipid lowering drugs) therapies developed in the last century led to the unprecedented prolongation of average human life duration. This spectacular achievement of modern medicine brought an unexpected effect in terms of demographic transition which now may be observed worldwide. In consequence, the number of people aged over 65 years, who in 2010 accounted for 8% of the total world population, is expected to rise up to approximately 16% by 2050 (World Health Organization, 2011). This process is particularly pronounced in Europe, where currently, those aged over 65 years constitute 19.2% of the European Union population, and this proportion is expected to increase up to 29.1% by 2080 (Eurostat, 2020).

The longer people live, the higher are their chances to develop medical conditions, in its most, non-communicable chronic diseases. Thus, prolonged life expectancy leads to longer years lived with multimorbidity defined by the World Health Organization (2008) as ‘the co‐occurrence of two or more chronic medical conditions in one person’. Current statistics prove that over three fourths of people aged over 65 years are subject to multimorbidity (Barnett et al., 2012; National Guideline Center, 2016; Stanley et al., 2018). Moreover, the therapeutic process in these patients is much more difficult for both healthcare professionals and for the patients themselves. This may lead to negative consequences and worse health outcomes in the long run (Maher et al., 2014).

As most chronic diseases typical for older adults are prevented and treated using pharmacotherapy, elderly patients are nearly automatically at high risk of multidrug therapy (Al-Dahshan et al., 2020; Panda et al., 2020). Thus, an older age is a risk factor for chronic polypharmacy (Wastesson et al., 2019). Numerous studies confirm that the highest prevalence of polypharmacy comes with age. A nationwide cohort study conducted in Sweden among individuals aged over 65 years found prevalence of polypharmacy at 44.0%, and the prevalence of extreme polypharmacy (defined as concurrent use of ten or more drugs) at 11.7% (Morin et al., 2018). Scottish data prove that around 35% of those aged over 85 years receive more than ten medicines (Barnett et al., 2012). A recent analysis of a large European cohort found polypharmacy to be present in 32.1% of citizens aged over 65 years, ranging from 26.3 to 39.9% across the studied countries (Midão et al., 2018). High prevalence of polypharmacy in elderly patients has also been reported outside Europe, e.g., in countries such as Brazil (Pereira et al., 2017) and the Unites States (Quinn and Shah, 2017).

On the other hand, as studies show, polypharmacy is not limited to the elderly. With more and more conditions being subject to effective pharmacotherapy (e.g., ADHD), it may be observed across all age groups, e.g., in pediatric population (Baker et al., 2019). Apart from age, several other drivers have been found to significantly affect the probability of polypharmacy. These include factors such as female gender, lower education, smoking, obesity and institutionalization (Haider et al., 2009; Haasum et al., 2012; Castioni et al., 2017; Carmona-Torres et al., 2018; Khezrian et al., 2020).

Out of the aforementioned enablers of polypharmacy, many are widespread in Poland. First of all, among European countries, Poland represents those with the fastest rate of the aging process (Eurostat, 2020). Moreover, for many years, it has also been a country with high use of drugs in general (Ozieranski and King, 2017). This is true despite the fact that Polish patients have to pay a lot for their medications. Similarly to many other European systems, Polish healthcare system is a public health insurance system based on a principle of social solidarity. Health services are provided free of charge to insured individuals (i.e., practically the whole population) by both public and private healthcare providers, and financed by the only national health payer–the National Health Fund (NHF, in Polish: *Narodowy Fundusz Zdrowia*). NHF also provides reimbursement of prescribed drugs. Nevertheless, most drugs are subject to out-of-pocket co-payment by patients, which vary across and within drug classes. Several drugs of crucial importance for particular therapies are available at a lump sum of PLN 3.20 (PLN - Polish zloty; approximately PLN 4.50 = EUR 1 as of December 2020), and some are free of charge. In the case of other medicines, patients pay 30, 50 or 100% of total drug costs out-of-pocket, depending on the effectiveness of the drug according to evidence-based criteria (e.g., homeopathic drugs are paid 100%). An extended list of free of charge drugs is available to citizens aged 75 years and more, and other selected groups of citizens, e.g., war veterans. On average, co-payment level for pharmacotherapy is still high in Poland, reaching more than 60% of original drug price (as of 2017) (Jahnz-Różyk et al., 2017).

This complex background of polypharmacy in Poland deserves careful attention, and implies adoption of effective preventive and corrective interventions. Of crucial importance is, however, an in-depth understanding of the prevalence of polypharmacy in both the general population, as well as across the age groups. This may help in better forecasting, planning and successful implementation of programs aimed at reducing the prevalence of polypharmacy. This type of studies has never been performed in Poland so far.

Therefore, the overall aim of this study was to determine prevalence of polypharmacy in Poland, and to assess the rate of this problem across various age groups, using real-world data for the general population.

## Methodology

### Data and Study Design

This was a retrospective analysis of the 2018 and 2019 anonymized aggregated drug dispensation data of NHF. The NHF database registers full information on dispensation of all drugs which are subject to reimbursement, no matter whether a particular prescription was issued by public or private healthcare provider. Starting from 2019, the database also registers information on non-reimbursed drugs dispensed according to prescriptions. It is possible due to the fact that since 2019 the community pharmacies have been obliged to generate and upload relevant records into the Medical Information System of Prescription Dispensation Documents (*Polish: System Informacji Medycznej Dokumentów Realizacji Recept*).

Thus, according to availability of data, we studied prevalence of polypharmacy caused by dispensation of reimbursed drugs only. Additional data collected for 2019 were used for supplementary calculation of polypharmacy prevalence based on both reimbursed and non-reimbursed drugs dispensed according to prescriptions in that period.

In order to avoid a bias of short-term therapies of no importance for chronic treatment, the analysis excluded medications from the following ATC (Anatomical Therapeutic Chemical) groups: A01 - Stomatological preparations, A06 - Drugs for constipation, D–Dermatologicals, J01 - Antibacterials for systemic use, J02 - Antimycotics for systemic use, J05 - Antivirals for systemic use, J06 - Immune sera and immunoglobulins, J07 – vaccines, P03 - Ectoparasiticides, including scabicides, insecticides and repellents and V–Various.

For the purpose of our analysis, polypharmacy was operationalized as taking five or more medications at the same time, according to the most commonly used approach, following the WHO report (World Health Organization, 2019). Six months’ long time period has been accepted as a basic framework of analysis. Accordingly, relevant numbers of drugs have been calculated according to the number of reimbursed drugs dispensed within six months from the first dispensation in the calendar year. For 2019 only, the numbers of both reimbursed and non-reimbursed drugs dispensed to the individuals have been calculated, and presented according to half-year periods, as well as for the whole calendar year.

For calculation purposes, the national population of Poland in 2018 was assumed to be 38,413,139, and in 2019 38,382,576, according to public statistics (Statistical Yearbook of Industry–Poland 2019).

### Ethics

Analyses of aggregated anonymized dispensation data do not involve ethical issues. Therefore, according to the policy of the Ethical Commission of the Medical University of Lodz, these data were not subject to the ethical approval procedure.

### Statistical Analyses

In descriptive statistics, both original numbers and the percentage rates calculated out of the total number of identified polypharmacy cases were presented, unless otherwise stated.

## Results

**FIGURE 1:**
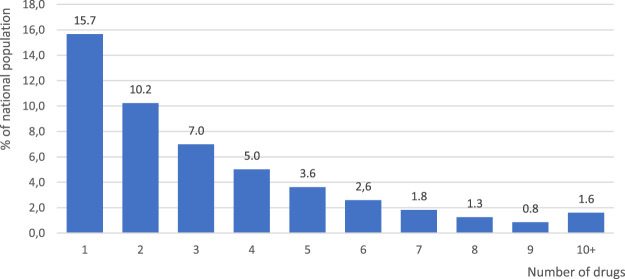
Distribution of the numbers of reimbursed drugs holding various active substances dispensed to patients according to prescriptions in Poland in 2018. Note: Numbers calculated for the drugs dispensed within six months from the first dispensation in the calendar year.

According to the analyzed NHF data, in 2018 a total of 23.3 million Polish citizens filled in their prescriptions for reimbursed drugs, and 19.1 million were dispensed medicines with ATC codes included in this study. The given number, on average, included 3.7 (+/−3.2) active substances, 4.8 (+/− 4.5) different drug EAN codes and 19.7 (+/−24.2) drug packages per patient per year. Figure 1 provides distribution of the number of reimbursed drugs with various active substances dispensed to patients in Poland in 2018 within six months from the first dispensation in that year. As many as 15.7% of Polish citizens were dispensed one drug only within that time frame, whereas 37.9% were dispensed up to four drugs. It is noteworthy that 1.6% of the national population was dispensed ten or more drugs with various active substances within six months from the first dispensation in 2018.

**FIGURE 2:**
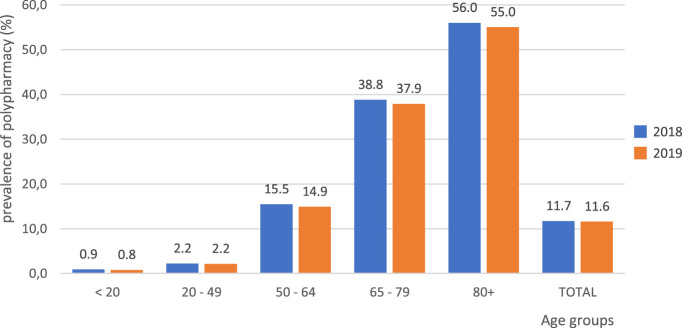
Prevalence of polypharmacy across age groups in Poland in 2018 and 2019. Note: Polypharmacy defined as dispensation of reimbursed drugs holding five or more active substances within six months from the first dispensation in the calendar year.

Prevalence of polypharmacy (calculated according to dispensation of the reimbursed drugs within six months from the first dispensation within a calendar year) and its distribution across age groups in 2018 and 2019 is presented in Figure 2. Polypharmacy defined that way was observed in 11.7%, and in 11.6% of Polish citizens in those two years, respectively. It is worth emphasizing that the older the age group, the higher was the prevalence of polypharmacy, reaching its highest value of 56.0% in those aged over 80 years in 2018, and 55.0% in the same age group in 2019. Altogether, among those aged over 65 years, prevalence of polypharmacy was 43.1% in 2018, and 42.1% in 2019. On the other hand, among those aged below 20 years, prevalence of polypharmacy was 0.9%, and 0.8% in 2018 and 2019, respectively.

Table 1 presents the number of patients by age group together with the number of active substances dispensed in 2019, as by the two halves of the year. The data relate to prescriptions filled in the above time period, for both drugs reimbursed and non-reimbursed. In the first half of 2019, in Poland there were almost 8.4 million citizens who were dispensed prescription drugs with at least five active substances. In this group, 4.3 million people, i.e., 51.8%, were individuals aged 65 years or more. In the second half of the year, there were slightly more (i.e., over 8.6 million) of such citizens, of whom 4.4 million, i.e., 50.8%, were people aged over 65 years. Prevalence of polypharmacy, defined as dispensation of five or more various drugs (including both reimbursed and non-reimbursed medications) within a half-year period, was 21.8%, and 22.4% for January‐June, and July‐December 2019 for the entire Polish population, respectively. Among those aged over 65 years, the relevant figures were 62.3%, and 62.9%, respectively.

**TABLE 1:** Age distribution of individuals dispensed various number of drugs holding various active substances according to prescriptions for both reimbursed and non-reimbursed drugs in half-year periods of 2019 in Poland.

No. of dispensed drugs	Jan-Jun 2019						Jul-Dec 2019					
	Age 0–64		Age 65+		Together		Age 0–64		Age 65+		Together	
	N	%	N	%	N	%	N	%	N	%	N	%
1	4,387 436	14.0	315,892	4.5	4,703 328	12.3	4,428 986	14.1	285,961	4.1	4,714 947	12.3
2	3,237 907	10.3	414,434	6.0	3,652 341	9.5	3,238 553	10.3	382,487	5.5	3,621 040	9.4
3	2,300 435	7.3	486,686	7.0	2,787 121	7.3	2,300 663	7.3	452,625	6.5	2,753 288	7.2
4	1,609 946	5.1	535,054	7.7	2,145 000	5.6	1,621 345	5.2	504,382	7.3	2,125 727	5.5
5	1,139 895	3.6	559,621	8.1	1,699 516	4.4	1,160 438	3.7	533,690	7.7	1,694 128	4.4
6	809,444	2.6	549,316	7.9	1,358 760	3.5	833,556	2.7	529,243	7.6	1,362 799	3.6
7	580,866	1.8	520,345	7.5	1,101 211	2.9	603,856	1.9	508,640	7.3	1,112 496	2.9
8	416,848	1.3	476,795	6.9	893,643	2.3	440,773	1.4	470,322	6.8	911,095	2.4
9	300,426	1.0	421,903	6.1	722,329	1.9	321,120	1.0	420,125	6.0	741,245	1.9
10	215,626	0.7	363,541	5.2	579,167	1.5	235,217	0.7	366,189	5.3	601,406	1.6
11	156,115	0.5	303,573	4.4	459,688	1.2	171,648	0.5	311,545	4.5	483,193	1.3
12	112,317	0.4	250,787	3.6	363,104	0.9	125,924	0.4	259,719	3.7	385,643	1.0
13	81,530	0.3	201,417	2.9	282,947	0.7	91,799	0.3	211,172	3.0	302,971	0.8
14	58,595	0.2	160,599	2.3	219,194	0.6	67,565	0.2	170,643	2.5	238,208	0.6
15	42,256	0.1	125,816	1.8	168,072	0.4	49,181	0.2	135,483	2.0	184,664	0.5
16	30,383	0.1	97,423	1.4	127,806	0.3	35,787	0.1	106,665	1.5	142,452	0.4
17	22,144	0.1	74,798	1.1	96,942	0.3	26,229	0.1	83,199	1.2	109,428	0.3
18	15,835	0.1	56,953	0.8	72,788	0.2	18,985	0.1	63,629	0.9	82,614	0.2
19	11,572	0.0	43,041	0.6	54,613	0.1	14,115	0.0	49,117	0.7	63,232	0.2
20+	31,380	0.1	125,313	1.8	156,693	0.4	41,081	0.1	150,864	2.2	191,945	0.5
TOTAL	**15,560 956**	**49.5**	**6,083 307**	**87.6**	**21,644 263**	**56.4**	**15,826 821**	**50.3**	**5,995 700**	**86.3**	**21,822 521**	**56.9**
5+	**4,025 232**	**12.8**	**4,331 241**	**62.3**	**8,356 473**	**21.8**	**4,237 274**	**13.5**	**4,370 245**	**62.9**	**8,607 519**	**22.4**
10+	**777,753**	**2.5**	**1,803 261**	**26.0**	**2,581 014**	**6.7**	**877,531**	**2.8**	**1,908 225**	**27.5**	**2,785 756**	**7.3**

Note: Percentage rates refer to the age groups, and the whole population, respectively. The same patient could appear in both half-year periods.

It is worth noting that a large group of individuals were dispensed as many as ten or more active substances in prescription drugs, which is often defined as extreme polypharmacy. In the first half of 2019, there were 2.6 million such patients, whereas in the second – 2.8 million. People aged over 65 years in the first half of the year accounted for 69.9% of the group (1.8 million), and in the second half of the year for 68.5% (1.9 million).

**FIGURE 3:**
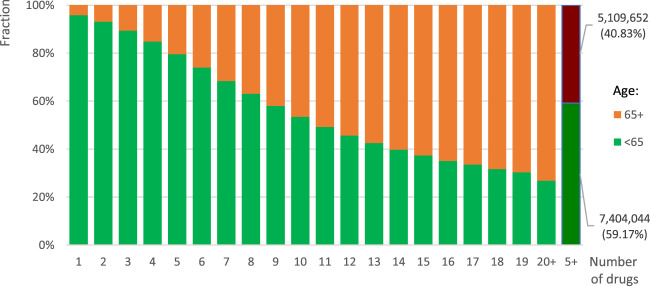
Age structure of individuals dispensed various number of drugs holding various active substances according to prescriptions for both reimbursed and non-reimbursed drugs in 2019 in Poland.

Figure 3 shows the age structure of patients depending on the number of various drugs dispensed in 2019. What is noteworthy is the fact that, along with the number of drugs dispensed, the percentage of elderly people increased up to 73.2% among those dispensed 20 or more drugs. Overall, among those who were dispensed five or more drugs, older adults aged 65 years or more accounted for 40.8%.

## Discussion

To the authors’ knowledge, this is the first large, nationwide population-based study on polypharmacy prevalence in Poland and one of the very few such wide-scale studies worldwide. Using real-world data, we have found high prevalence of polypharmacy reaching 11.7% of the total Polish population in 2018, and 11.6% in 2019, when the reimbursed drugs only were considered. This number rose up to 22.4% for the second half of 2019, if the data on dispensing reimbursed and non-reimbursed drugs were analyzed collectively.

These findings correspond with results of studies performed in other countries, despite several differences in methodology (e.g., different time frame, or number of dispensed drugs defining polypharmacy). Out of studies using threshold of ≥5 drugs, two assessed polypharmacy in Sweden and found to involve 11.1%, and 19.0% of the total population in 2008 and 2014, respectively (Hovstadius et al., 2010; Zhang et al., 2020). A study analyzing data from pharmacy claims of 1.7 million citizens found polypharmacy in 17% of Swiss population in 2013 (Blozik et al., 2013). A Danish study, defining polypharmacy as >5 different medicines dispensed in the first half of 2016, found its prevalence in 12% of the entire population (Kornholt and Christensen, 2020). A study in a population of one of the Japanese prefectures revealed prevalence of polypharmacy (defined as prescribing of six or more drugs per month) at the level of 20.0% (Amano et al., 2020). In fact, studies smaller in data size observed similar results. Polypharmacy was observed among 15.8% of Spanish adults (Niclós et al., 2018). Out of 180,815 Scottish adults studied, 16.9% were receiving from four to nine medications prescribed regularly, and 4.6% were receiving ten or more medications (Payne et al., 2014).

Not surprisingly, we have found prevalence of polypharmacy increasing with age, with relevant percentage rates exceeding one third in the age group 65–79 years, and reaching 56.0% for those aged over 80 years in 2018 when analyzing reimbursed drugs only. Altogether, for the entire group of elderly citizens (i.e., those aged over 65 years) we observed polypharmacy prevalence exceeding 40% in both studied years when analyzing reimbursed drugs only. However, relevant numbers rose to nearly two thirds for the elderly when analyzing both reimbursed and non-reimbursed drugs (62.3% and 62.9%, for the first and second half of 2019, respectively).

In this case our results are again similar to those obtained in other countries, proving polypharmacy particularly prevalent in the elderly. In Sweden, polypharmacy rates for age groups <60, 60–69, 70–79, 80–89 and over 90 years were 8.5, 35.9, 54.8, 73.0 and 79.6%, respectively in 2014 (Zhang et al., 2020). In Italy, polypharmacy was observed in 39.4% of the elderly in 2007 (Slabaugh et al., 2010), in Switzerland in 41% in 2013 (Blozik et al., 2013), in Denmark in 51% of those aged over 75 years in 2016 (Kornholt and Christensen, 2020). Polypharmacy (defined as use of five or more medications in the last two weeks), and excessive polypharmacy (defined as use of ten or more medications in the last two weeks) was found in 21.9%, and 0.6% community-dwelling Spanish older adults, respectively (Carmona-Torres et al., 2018). In a recent European study, polypharmacy was identified in 32.1% of people aged over 65 years in Europe, ranging from 26.3 to 39.9% across the studied countries (Midão et al., 2018). In Poland, this ratio was already shown to be higher and amounted to approximately 33.8% (Midão et al., 2018).

It is noteworthy that in other age groups, e.g., among those aged below 20 years, we observed polypharmacy to be less prevalent, yet present, at the level not exceeding 1%. A recent scoping review proved high prevalence of polypharmacy in pediatric patients, ranging from 0.9 to 98.4% (median 39.7%) (Baker et al., 2019). However, that review defined pediatric polypharmacy as taking more than one medication (Feinstein et al., 2015), whereas in our study, a uniform operational definition of polypharmacy was used for all age groups, based on concurrent use of five or more drugs.

An important clinical implication of our study is that polypharmacy is highly prevalent in Poland. Since the elderly were found to represent a majority of patients exposed to polypharmacy, particular attention should be focused on these patients. However, polypharmacy was proven to be a problem not limited to the elderly only. Being aware of this fact, clinicians should pay much more attention to the issue of polypharmacy across all age groups.

A recent WHO report on polypharmacy underpins this problem, and urges different countries to take early priority action to protect patients from harmful effects of polypharmacy by implementing dedicated programs (World Health Organization, 2019). Unfortunately, a search for polypharmacy management programs, undertaken recently within the framework of the SIMPATHY project, revealed existence of such dedicated initiatives in five out of nine assessed countries only (McIntosh et al., 2018). Moreover, no official program of that kind was identified in Poland (Stewart et al., 2017). To the authors’ knowledge, the scenario in this area has not changed in Poland until now. Under these circumstances, a comprehensive, policy-driven, and evidence-based approach to management of inappropriate polypharmacy which was introduced in Scotland is particularly interesting since it may serve as an example of good practice (Wilson et al., 2015). Another crucial issue is, however, reaching individual prescribers as studies in many countries proved high variability of polypharmacy prevalence across primary care centres (Franchi et al., 2013; Sinnige et al., 2016).

The WHO report provides several practical tips on how to reduce the burden of polypharmacy problem, and showcases several European projects focused on obtaining the goal. In particular, the WHO report encourages use of medication reviews, i.e., structured evaluations of a patient’s medications, with the aim of optimizing application of medicines and improving health outcomes. This entails detecting drug-related problems and recommending interventions. Additionally, it advocates the concept of deprescribing, i.e., the process involving tapering, stopping, discontinuing, or withdrawing drugs, with the goal of managing polypharmacy and improving outcomes. (World Health Organization, 2019). So far, several clinical algorithms and guidelines have been published in order to reduce inappropriate prescribing and manage polypharmacy (Muth et al., 2019; Lee et al., 2020). The most commonly acknowledged ones are instruments such as Beers and STOPP/START criteria. In brief, the main assumptions are that while managing polypharmacy, a clinician screens the patient’s drug list for repetitions of the same substance under different market names, drug-drug interactions, drug-disease interactions, unnecessary drugs and drugs that in the patient’s current clinical state may be replaced with potentially less harmful ones. In primary care, it is recommended to perform a “brown-bag review” (a review of all the medications, including OTC drugs and dietary supplements) once a year (Nathan et al., 1999).

A certain limitation of this study is that we could not seek for possible correlations between the number of conditions a particular patient was diagnosed with, their characteristics, or formally diagnosed multimorbidity, and the individual exposure to polypharmacy. Similarly, we could neither investigate the rationales for identified polypharmacy cases, nor dichotomize them into appropriate and inappropriate ones. It was not possible due to the characteristics of data that were available for our analysis, i.e., dispensation data only. To overcome these limitations, an access to full medical history of each patient would be necessary. Unfortunately, a nationwide electronic health record system has not been launched yet in Poland, which makes comparisons between conditions diagnosed and drugs prescribed and dispensed for individuals practically impossible. Thus, we may only hypothesize that multimorbidity must have had an effect on polypharmacy prevalence in the studied Polish population. Numerous data show that the greater the number of conditions a patient is diagnosed with, the higher is the probability of polypharmacy (Slabaugh et al., 2010; Payne et al., 2014; Khezrian et al., 2020).

Another limitation of our analysis was that, being based on dispensing data, it could not assess patients’ real daily exposure to drugs. This, in fact, shall be taken into consideration while interpreting the study results, as adherence is a major factor contributing to the number of drugs and individual doses that the patient uses. The actual degree of drug use is modified by patient adherence, which varies from over- to underuse of prescribed drugs. Fortunately, the data analyzed by us, i.e., dispensation data, are not biased by primary non-adherence, which was recently found, in other studies conducted by our group, to reach the overall level of 20.8% (Kardas et al., 2019), with some drug groups reaching even higher values (e.g., 31.3% in antihistamines (Kardas et al., 2020)). On the other hand, real-world drug use is affected by secondary non-adherence, which in Poland in some cases reached the level of over 80% (Kardas, 2011). Of course, secondary non-adherence most often leads to underuse of drugs. However, not only the opposite might be true, but also postponed doses taken cumulatively may expose a patient to increased risk of negative consequences of polypharmacy, e.g., drug-drug interactions.

An obvious limitation of our study comes with the fact that the scope of the analyzed drugs was narrowed down to prescription drugs only, and as in the case of 2018 results, only reimbursed drugs were included. In fact, polypharmacy is a problem which might be caused by various sort of remedies, including non-reimbursed prescription drugs, as well as over-the-counter (OTC) drugs and dietary supplements which are often overused. On the other hand, it is worth emphasizing that in the case of an analysis on drug intake by patients, focused on such aspects as adherence or polypharmacy levels, results based on administrative data are considered a reliable source of information, as compared to surveys or patient reports, which are subject to e.g., recall bias (Schmier and Halpern, 2004).

Finally, it should be kept in mind that our study was based on a nationwide dispensation database, and only a few drug groups of minor importance were excluded from analysis for practical reasons. Thus, we believe that the selection bias of our results was as low as possible.

This study has also a number of strengths. It provides new, important information. With use of high-quality, complete nationwide data we have assessed prevalence of polypharmacy in Poland, as well as its distribution across various age groups. This is one of the very few studies which clearly show that polypharmacy, peaking in the elderly, occurs in fact across all ages. Thus, we believe that future studies may cover this problem in a wide age spectrum, and be focused at identification of major risk factors.

Moreover, our results point to the benefit of using high quality real-world data for polypharmacy assessment. It is noteworthy that when the new method of data collection created an option for the analysis of dispensation of both reimbursed, and non-reimbursed drugs, the observed prevalence of polypharmacy nearly doubled, increasing from 11.6%, to 22.4% within the same year 2019. This undoubtedly proves advantages of using more comprehensive data for an analysis.

The results of the study also lay the foundation for interventions aimed at lowering the prevalence of polypharmacy in Poland. With polypharmacy coming with age, and a continuous trend of an increasing fraction of older adults in Polish society, the elderly become the primary target for these initiatives. However, our results undoubtedly point to the fact that not only the elderly, but also many middle-aged and younger patients should be carefully targeted for that problem. With the introduction of the nationwide Electronic Health Record system in Poland, which is scheduled for mid-2021, this goal could become much easier to achieve. Further developments of national eHealth solutions, and digitization of the healthcare system could also help this.

## Conclusion

This study was the first large, nationwide assessment of polypharmacy prevalence in Poland. Using real-world data, it confirmed high prevalence of polypharmacy affecting one fifth of the national population. Peaking in the elderly, polypharmacy was found in each age group. These findings lay the foundation for future interventions aimed at lowering the burden of this problem in Poland. A broader implementation of eHealth solutions may help to exploit the full potential of real-world data, and implement these interventions at an individual patient level.

## Data Availability

The data analyzed in this study is subject to the following licenses/restrictions: The data that support the findings of this study are available from NHF (data owner). Restrictions apply to the availability of these data, which were used under license for this study. Data are available from the authors with the permission of NHF. Requests to access these datasets should be directed to pkardas@csk.am.lodz.pl.
